# Temporal Patterns and Environmental Correlates of Macroinvertebrate Communities in Temporary Streams

**DOI:** 10.1371/journal.pone.0142370

**Published:** 2015-11-10

**Authors:** Paul K. Botwe, Leon A. Barmuta, Regina Magierowski, Paul McEvoy, Peter Goonan, Scott Carver

**Affiliations:** 1 School of Biological Sciences, University of Tasmania, Hobart, Tasmania, Australia; 2 Department of Ecology, Environment and Evolution, La Trobe University, Melbourne, Victoria, Australia; 3 Department of Environment, Water and Natural Resources, Adelaide, South Australia, Australia; 4 South Australia Environment Protection Authority, Adelaide, South Australia, Australia; Argonne National Lab, UNITED STATES

## Abstract

Temporary streams are characterised by short periods of seasonal or annual stream flow after which streams contract into waterholes or pools of varying hydrological connectivity and permanence. Although these streams are widespread globally, temporal variability of their ecology is understudied, and understanding the processes that structure community composition in these systems is vital for predicting and managing the consequences of anthropogenic impacts. We used multivariate and univariate approaches to investigate temporal variability in macroinvertebrate compositional data from 13 years of sampling across multiple sites from autumn and spring, in South Australia, the driest state in the driest inhabited continent in the world. We examined the potential of land-use, geographic and environmental variables to predict the temporal variability in macroinvertebrate assemblages, and also identified indicator taxa, that is, those highly correlated with the most significantly associated physical variables. Temporal trajectories of macroinvertebrate communities varied within site in both seasons and across years. A combination of land-use, geographic and environmental variables accounted for 24% of the variation in community structure in autumn and 27% in spring. In autumn, community composition among sites were more closely clustered together relative to spring suggesting that communities were more similar in autumn than in spring. In both seasons, community structure was most strongly correlated with conductivity and latitude, and community structure was more associated with cover by agriculture than urban land-use. Maintaining temporary streams will require improved catchment management aimed at sustaining seasonal flows and critical refuge habitats, while also limiting the damaging effects from increased agriculture and urban developments.

## Introduction

Temporary streams, characterised by the repeated onset and cessation of flow are widespread globally and common in agricultural and urban landscapes [1–3]. Macroinvertebrate communities in these streams are thought to be largely driven by flow variability, and this temporal variability is an important, but understudied, aspect of their ecology. Although our understanding of spatio-temporal variation in patterns of macroinvertebrate community structure in permanent streams has greatly improved [4], few studies have examined temporal variability of macroinvertebrate communities in temporary streams [5].

Temporary streams present challenges for assessing environmental condition and impacts owing to their inherent variability. For example, stream condition is often assessed with predictive models based on macroinvertebrate occurrence at reference sites that are only minimally affected by human disturbance. This is the case in Australia where the observed/expected predictive models developed as part of the Australian River Assessment System [AUSRIVAS] [6, 7] are based on the assumption that macroinvertebrate assemblages are relatively spatio-temporally consistent in the absence of anthropogenic perturbation, and that sampling sites are suitably similar (or undisturbed enough, in the case of reference sites) to allow robust comparison [8]. However, these ideal conditions are difficult to define in highly seasonal dry-land streams, and demonstrating that changes in macroinvertebrate assemblages are caused by anthropogenic disturbance is difficult when the natural variability of assemblages in such systems is unknown [9]. Furthermore, Larned, Datry [10] argued that water managers usually mis-manage temporary streams by applying perennial stream management principles, thus leading to potentially erroneous decisions about best management practices.

Despite global concern over current and future land-use impacts, little attention has been given to the effects of land-use on temporary streams [11], primarily because of the episodic nature of these streams [12]. Reviews by Johnson and Host [13], Steel, Hughes [14] and Allan [15] have highlighted the need for an improved understanding of the mechanisms by which land-use and related environmental variables alter stream biota and habitats.

In this study, we explored relationships of macroinvertebrate community composition to land-use, geographic and environmental variables in the Mount Lofty Ranges and Kangaroo Island; two warm temperate regions in South Australia with a largely Mediterranean climate. Temporary streams are abundant in South Australia [16] and include a spectrum of annual flow-cessation regimes in a state where land-use varies in its intensity. Our study spanned multiple sites sampled in two seasons for 13 years, with sites varying in terms of the proportion of land-uses in their upstream catchments. We aimed to: (i) explore the temporal variability and trajectories of macroinvertebrate composition; (ii) examine the potential of land-use, geographic and environmental variables to predict macroinvertebrate assemblages and; (iii) identify indicator taxa that are correlated with gradients of specific land-uses, environmental and geographic variables.

## Material and Methods

### Ethics Statement

This research did not involve vertebrates or cephalopods and therefore was not required to be approved by the Animal Ethics Committee of the National Parks and Wildlife Division of the South Australian Department of Environment and Heritage, which complies with the Australian Code of Practice for the Care and Use of Animals for Scientific Purposes (8^th^ Edition, 2013), the Prevention of Cruelty to Animals Act 1985 (South Australia), and the Australian Code for the Responsible Conduct of Research (2007). All sites sampled (see S3 Table) were on private state or crown lands and permits were not required. The Department of Environment, Water and Natural Resources (South Australia) who manage crown lands confirmed that no permits were required for access to crown land. Land title details can be found on the South Australian Integrated Land Information System (‘SAILIS’ www.sa.gov.au/topics/housing-property-and-land/land-services-industry/sailis). Most taxa sampled in this research were identified to species and no taxa were listed as endangered or protected in Australian state or federal legislation.

### Study area and macroinvertebrates sampling

**Fig 1:**
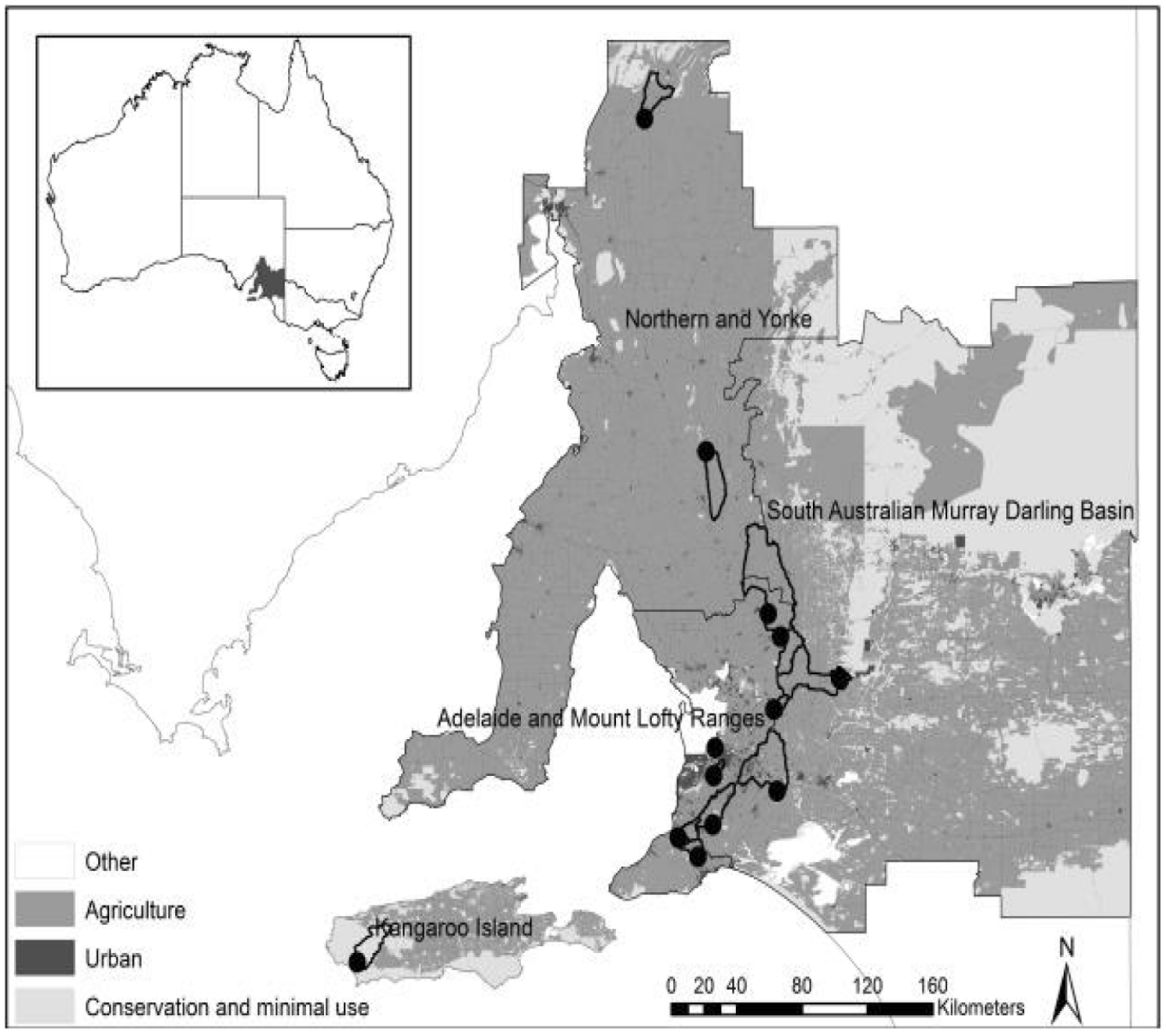
Map of South Australia, showing the distribution of major land-uses and the sampling sites. Circles represent study sites; heavy black lines represent catchment area upstream of study sites; grey lines represent coastline and state borders; thin black lines represent boundaries to NMR regions. Land-use South Australia layer was sourced from Australian Natural Resources Data Library and their classifications were based on the Australian Land-use and Management (ALUM) classification.

Our study sites were in four of the eight Natural Resource Management (NRM) regions in South Australia. Sites include the Adelaide and Mount Lofty NRM region (Western MLR = 7 sites including Hindmarsh, Torrens, North Para, Myponga and Light rivers, First and Scott creeks), Murray-Darling NRM region (3 sites including Finnis, Marne and Bremer rivers), Northern and Yorke NRM (2 sites including Hill and Kanyaka rivers) and Kangaroo Island NRM (1 site from Rocky river) (Fig 1). The macroinvertebrate samples analysed here form part of the Australian Rivers Assessment System (AUSRIVAS) [17] of which South Australia has been part since 1994. The database includes a substantial, standardised and consistent record on benthic macroinvertebrates and a large number of environmental variables. Annual sampling was conducted in two seasons (autumn and spring), to represent the extremes of variation in physicochemical properties such as temperature, dissolved oxygen and flow, which likely drive differences in biological productivity and biodiversity. We used data collected annually for 13 years from 1994 to 2007 (except 1996 owing to hiatus in national program funding).

Macroinvertebrates were collected using standardised AUSRIVAS protocols which consisted of sampling representative 5 m^2^ area of pool habitats within each 100 m study site using a 250 μm mesh square dip net. Sampling involved vigorously kicking the substrate and sweeping the net over a total bank length of 10 m using sequential short sweeping movements at right angles to the bank and, sweeping under overhanging or emergent vegetation [17]. Collected macroinvertebrates were preserved in ethanol on site, transported to the laboratory, and subsampled (where 10% of the samples were counted and identified using light and dissecting microscopes), and the residue scanned for rare taxa [7, 17]. This approach ensured observer bias was minimised when counting individuals compared to alternative live-pick approaches included in the AUSRIVAS protocols and it also provided an accurate estimation of the abundance of cryptic taxa. Taxa were identified to the lowest taxonomic level, given available keys, life-history stage and condition. This was most often to genus or species level except for Hydracarina (mites), and some Oligochaeta (worms). Voucher specimens of all taxa were retained as a reference collection at the South Australia Museum and Australian Water Quality Centre (AWQC).

### Land use and environmental information

To calculate land-use in the upstream catchment of each site, we used the GIS “Land use South Australia” layer which is based on remote-sensing satellite data [Australian Natural Resources Data Library [18]]. Areas of South Australia mostly used for agriculture have been mapped at 1:25 000, whereas the remaining areas have been mapped at 1:100 000. Land-use categories were based on the Australian Land-use and Management (ALUM) classification (based on remote-sensing satellite data compiled in, 2003). Land-use information was derived as percentage (%) of catchment area upstream from each sampling site. We aggregated several land use categories per site (see supporting S1 Table for details). Existing land-use data [[18]; Fig 1] showed that conservation areas (formal and informal reserves) comprised 38% of the land area. Agriculture (mostly cattle and sheep grazing) of varying types and intensity, and to a lesser extent dryland cereal cropping accounted for 53% of land area. The remaining 3% comprised rural residential and urban uses.

Conductivity, dissolved oxygen and pH were measured *in situ* using calibrated water quality meters. At each site, the physical habitat was characterized as the cover of sand, silt and clay on a scale (0% = no cover, 1–25% = little cover, 26–50% = some cover, 51–75% = moderate cover, 76–100% = extensive cover). Monthly average of estimated local discharge (runoff + drainage) at time of sampling for each stream were sourced from the Australian Water Availability Project (AWAP) [19, 20].

### Statistical analysis

#### Univariate measures

Analyses were conducted on multivariate (site by taxa abundance matrix) and univariate response variables. Any taxa with fewer than 5 individuals across the 13-year sampling period were considered rare and were excluded from this analysis. The univariate measures were selected based on [21] and included: (i) Margalef’s richness, defined as the total number of different species represented or total number of individuals of all species in the sample. We calculated Margalef’s richness using the formula *d* = (*S*-1) / log(*N*), where, *S* is the number of species and *N* is the abundance or total number of individuals [22]; (ii) Simpson’s diversity index, measures the probability that two individuals randomly selected from a sample will belong to the same species. We calculated Simpson’s index as *D* = 1 − ∑(n_*i*_ × (n_*i*_ − 1) / (*N* × (*N* − 1)), where *n*_*i*_ is the total number of macroinvertebrates of a particular species (the *i*th taxon) and *N* is the total number of macroinvertebrates of all species [23] and; (iii) Pielou’s evenness calculated as *J* = *H* / log(*S*), where, *H* is Shannon-Weiner diversity and *S* is the total number of species [24]. All diversity indices were computed using PRIMER-E (v6.1.16) [25], and *R* version 3.2.0 [26] with the vegan package [27].

### Aim 1: Temporal changes in macroinvertebrate composition

To explore temporal changes in assemblage composition with three factors (site, season and year), permutational multivariate analysis of variance (PERMANOVA) [28] based on Bray-Curtis similarity of fourth-root transformed data [29] was used. Site was a nested factor within season and year, and formal testing was undertaken using the PERMANOVA+ (v1.0.6) software extension to PRIMER [25]. Hybrid multidimensional scaling (semi-strong hybrid MDS, threshold = 0.9) [30] was used to visualize temporal changes in macroinvertebrate assemblages within sites and seasons using the “vegan” package [27]. We tested for time (annual) trend of the assemblage trajectories using the seriation test of the RELATE routine in PRIMER-E [25, 31]. A permutation test (9999 permutations) was used to evaluate significance. The seriation test is based on the assumption that adjacent sample years tend to be closest together in terms of their communities than sample years which are further apart in time [25, 31]. The test examines the correlations between dissimilarity of communities and time. If |0.8 ≤ ρ ≤ 1.0| (where, ρ is the Spearman’s correlation), then there is a clear trend in the trajectories of the community composition [28], which will be evident by a straight line (a trajectory) in the hybrid MDS plot. Seasonal and annual changes in univariate indices were analysed with generalized linear modelling (GLM) in R.

### Aim 2: Relationship of macroinvertebrate community composition to environmental, geographic and land use predictors

We used land-use, geographic and environmental variables to examine which variables may be correlated with macroinvertebrate community composition. To explore these relationships, we first examined co-linearity among normalized geographic, environmental and land-use variables using Spearman’s correlation coefficient (ρ) and scatter plot matrices to eliminate co-linear variables and reduce redundancy (see supporting S2 Table for details). Variables with the greatest potential ecological importance were used as surrogates for those variables with which they were highly correlated (|ρ ≥ 0.9|) [28]. Distance from source (DFS) and conservation and minimal use (consvMin) (S1 Table) were excluded from the analysis because they were highly correlated with catchment area and percentage cover by agriculture respectively. Catchment area or stream size was chosen over DFS because macroinvertebrate species richness has been cited to exhibit strong relationships to catchment area [32, 33]. Agriculture was chosen over consvMin because gradients of intensity of agricultural land-use were logically more likely to be associated with changes in assemblage structure from reference/natural condition. Therefore, 12 out of 14 initial candidate variables were used for this analysis. We included geographic location variables (latitude and longitude) to capture any biogeographic variation in community composition across the large spatial extent of this study.

For the multivariate analysis, a distance-based linear model (DistLM) [25, 34] with stepwise regression as selection procedure, using Akaike Information Criterion (AIC) as the selection criterion was used to derive the most parsimonious models predicting macroinvertebrate communities, and for the distance-based redundancy analysis (dbRDA) models. The DistLM enabled us identify predictor variables (on the normalised scale) that contributed significantly to the temporal patterns observed in the assemblage structure as well as determine how much variation was explained by each predictor. The dbRDA plot enabled us visualize the relative contributions of each of the predictor variables on the assemblage structure [35–37]. For the univariate analysis, a stepwise regression methods using AIC as the selection criterion was again used to derive the most parsimonious models for each univariate measure. Diagnostic analysis using Variance inflation factors (VIF) were employed to examine how much multicollinearity (correlation between predictors) exist in the multiple regression analysis. None of the VIF inspected exceeded 2.5, so the partial regression coefficients likely provided reliable estimates of effects of each predictor variable while holding the effects of all other variables constant [38].

### Aim 3: Candidate taxa that correlate with gradients of land-use, environmental or geographic variables

To examine which taxa were correlated to gradients of the significant predictors identified by DistLM in the Aim 2, we used the BVSTEP (Best Subset of Environmental Variables with maximum Correlation with Community Dissimilarities) procedure in PRIMER [25, 28]. This procedure finds subsets of taxa (vulnerable and opportunistic taxa) which are best correlated with the patterns in the predictor variables (on a distance matrix between predictors) [39]. A permutation test (9999 permutations) was used to evaluate the significance of the results. Individual Spearman’s rank correlations (ρ) were then used to evaluate the direction and strength of the relationships between each taxon and each predictor variable. We define indicator taxa as those which were significantly (*P* ≤ 0.05) correlated with the predictor variables.

## Results

We collected 338 samples which comprised 173,149 individuals from 840 taxa. Autumn samples comprised 66,503 individuals while 106,646 individuals were recorded in spring.

### Aim 1: Temporal change in assemblage composition

**Fig 2:**
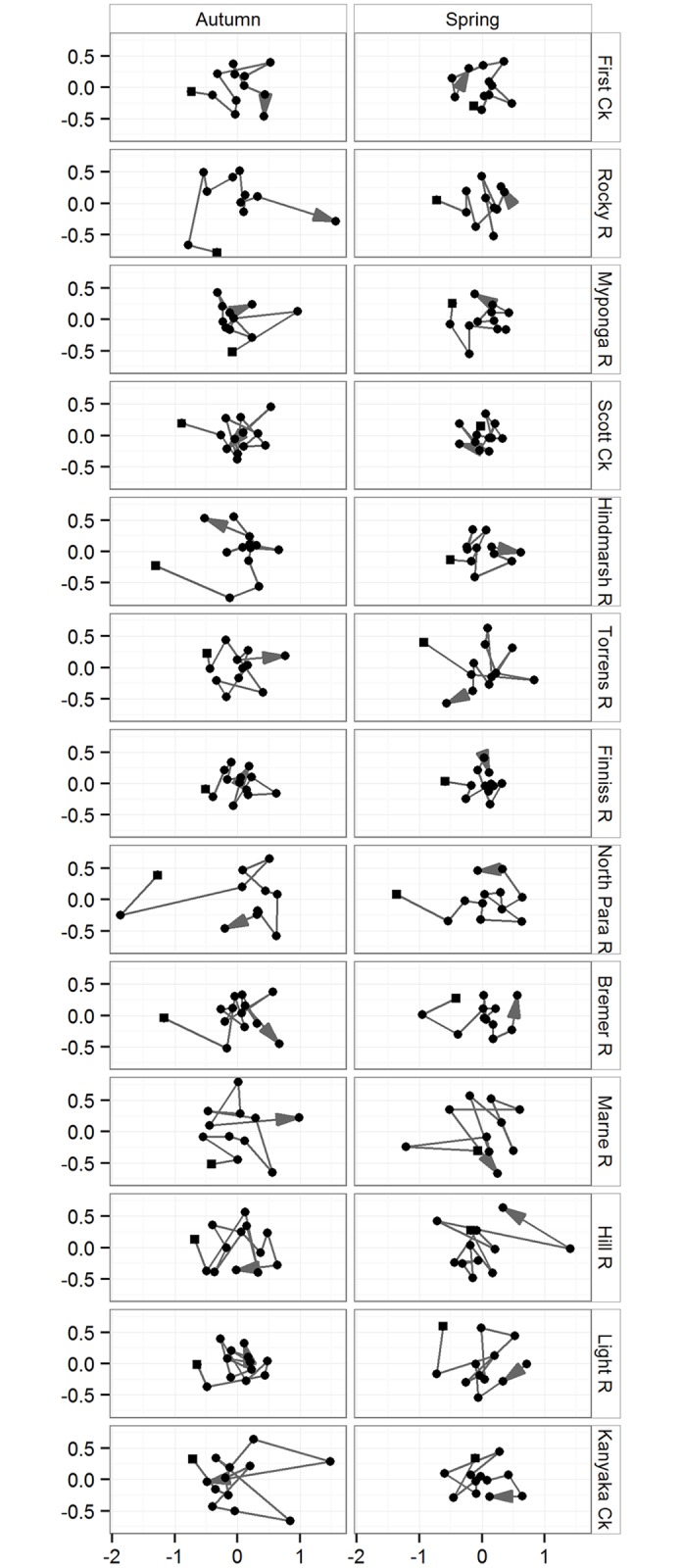
Unconstrained ordination plots of macroinvertebrates in autumn and spring. Unconstrained (semi-strong hybrid MDS) ordination plots of macroinvertebrates (individual sites across years) based on Bray-Curtis similarity of 4^th^ root abundance data in autumn and spring. Sites names with “Ck” and “R” represent creeks and rivers respectively. The lines connecting the dots represent trajectories of assemblage structure across the years. Square symbols indicate the start of the trajectory and the arrow head indicates the end of the trajectory. The scale represents dissimilarity of sites.

Multivariate analysis showed that macroinvertebrate community composition differed significantly among sites (Table 1), as did the shape of their trajectories (Table 2, Fig 2). Within sites, macroinvertebrate communities varied between autumn and spring and differed among the years (Table 1), but there was no clear trend in their trajectories within sites across years (as indicated by ρ < 0.8 from the RELATE procedure) (Table 2, Fig 2). There was no indication that sites changed in any consistent way among years (Fig 2).

**Table 1:** Results of permutational multivariate analysis of variance (PERMANOVA). *df* represents degrees of freedom. Bold numbers indicate significant *P*-values.

	*df*	*F*	*P*
Site	12	4.02	**< 0.001**
Season(Site)	13	2.48	< **0.001**
Year(Site)	155	1.40	**< 0.001**
Residual	136		

**Table 2:** RELATE results (ρ and *P*-value) reported for seriation of macroinvertebrate composition at each site for each season. Sites names with “Ck” and “R” represent creeks and rivers respectively. ρ signifies Spearman’s correlations in the seriation test; if |0.8 ≤ *ρ* ≤ 1.0|, then there is a clear trend in the trajectories of the community composition [28].

Site	Autumn	Spring
	ρ	ρ
First Ck	0.41	0.30
Rocky R	0.55	0.38
Myponga R	0.34	0.48
Scott Ck	0.16	0.22
Hindmarsh R	0.45	0.41
Torrens R	0.37	0.32
Finniss R	0.37	0.35
North Para R	0.47	0.49
Bremer R	0.34	0.56
Marne R	0.29	0.12
Hill R	0.17	0.27
Light R	0.32	0.21
Kanyaka Ck	0.12	0.20

Univariate analysis based on species richness, evenness and diversity indicated significant differences among sites (Table 3). Richness did not differ across seasons but varied significantly across the years. Within sites, Evenness was higher in autumn (0.63 ± 0.01) than in spring (0.58 ± 0.01) but did not differ among years. Diversity was also higher in autumn (0.76 ± 0.01) than in spring (0.72 ± 0.02), but also did not differ among years. The GLMs explained more of the variation in richness than evenness or diversity (Table 3).

**Table 3:** Results of general linear models for the relationships of the biodiversity indices to site, season (site nested within season) and year (site nested within year).

	*df*	MS	*F*	*P*
**Richness** (*R* ^2^ = 0.528)				
Site	12	37.74	19.69	**< 0.001**
Season(Site)	13	2.04	1.06	0.392
Year(Site)	13	8.94	4.67	**< 0.001**
Residuals	278	1.92		
**Evenness** (*R* ^2^ = 0.355)				
Site	12	0.16	9.06	**0.001**
Season(Site)	13	0.05	2.93	**< 0.001**
Year(Site)	13	0.01	0.46	0.947
Residuals	278	0.02		
**Diversity** (*R* ^2^ = 0.310)				
Site	12	0.16	6.83	**< 0.001**
Season(Site)	13	0.06	2.84	**0.001**
Year(Site)	13	0.01	0.39	0.972
Residuals	278	0.02		

*df* represents the degrees of freedom for the sources of variation. Bold numbers indicate significant *P*-values.

### Aim 2: Relationships of macroinvertebrate community composition to environmental, geographic and land-use predictor variables

**Fig 3:**
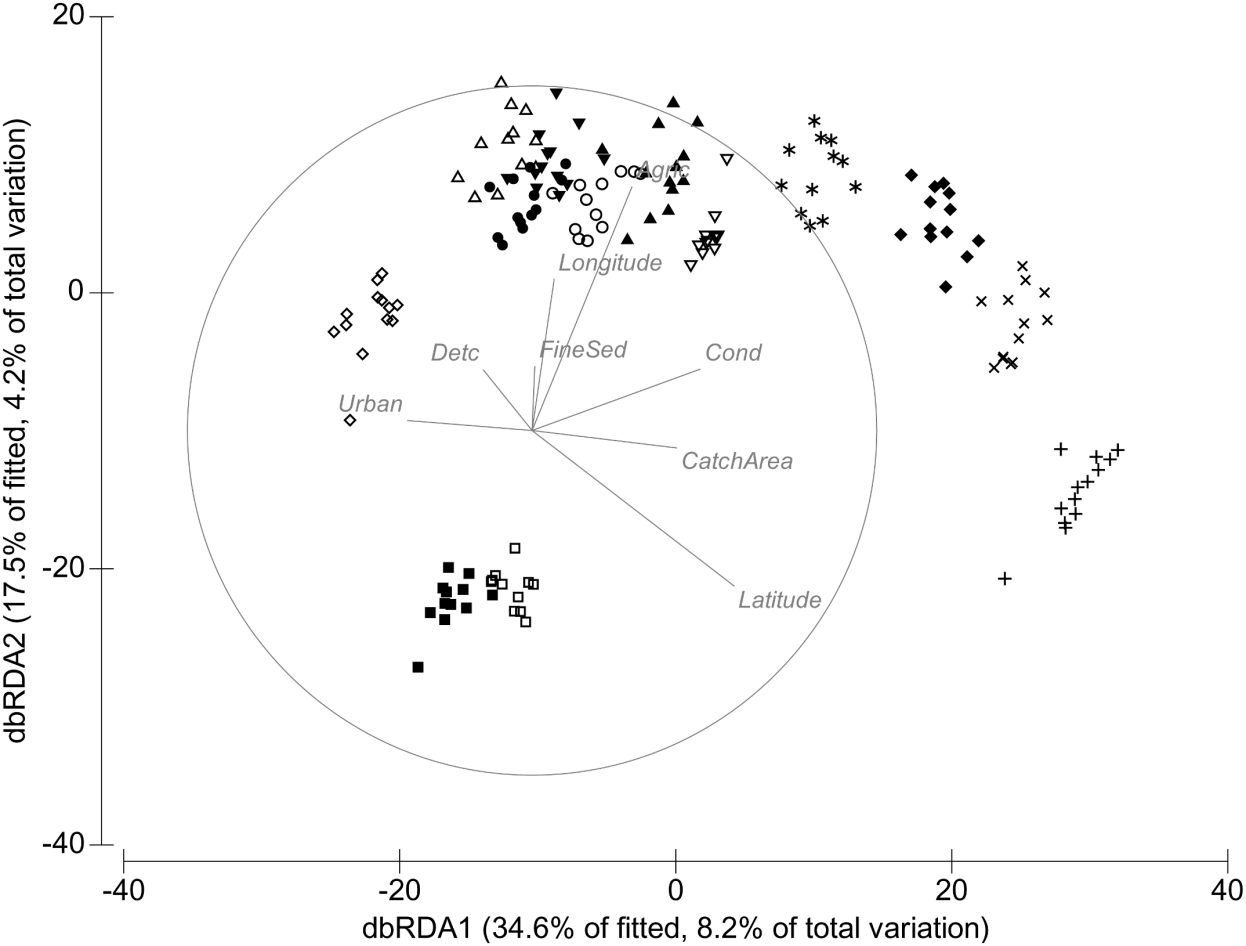
Distance-based redundancy analysis (dbRDA) of macroinvertebrate samples in autumn. Distance-based redundancy analysis (dbRDA) of macroinvertebrate samples in autumn, overlaid with normalised predictor variables (based on distLM analysis in Table 4). Abbreviations for predictor variables are listed in S1 Table.

**Fig 4:**
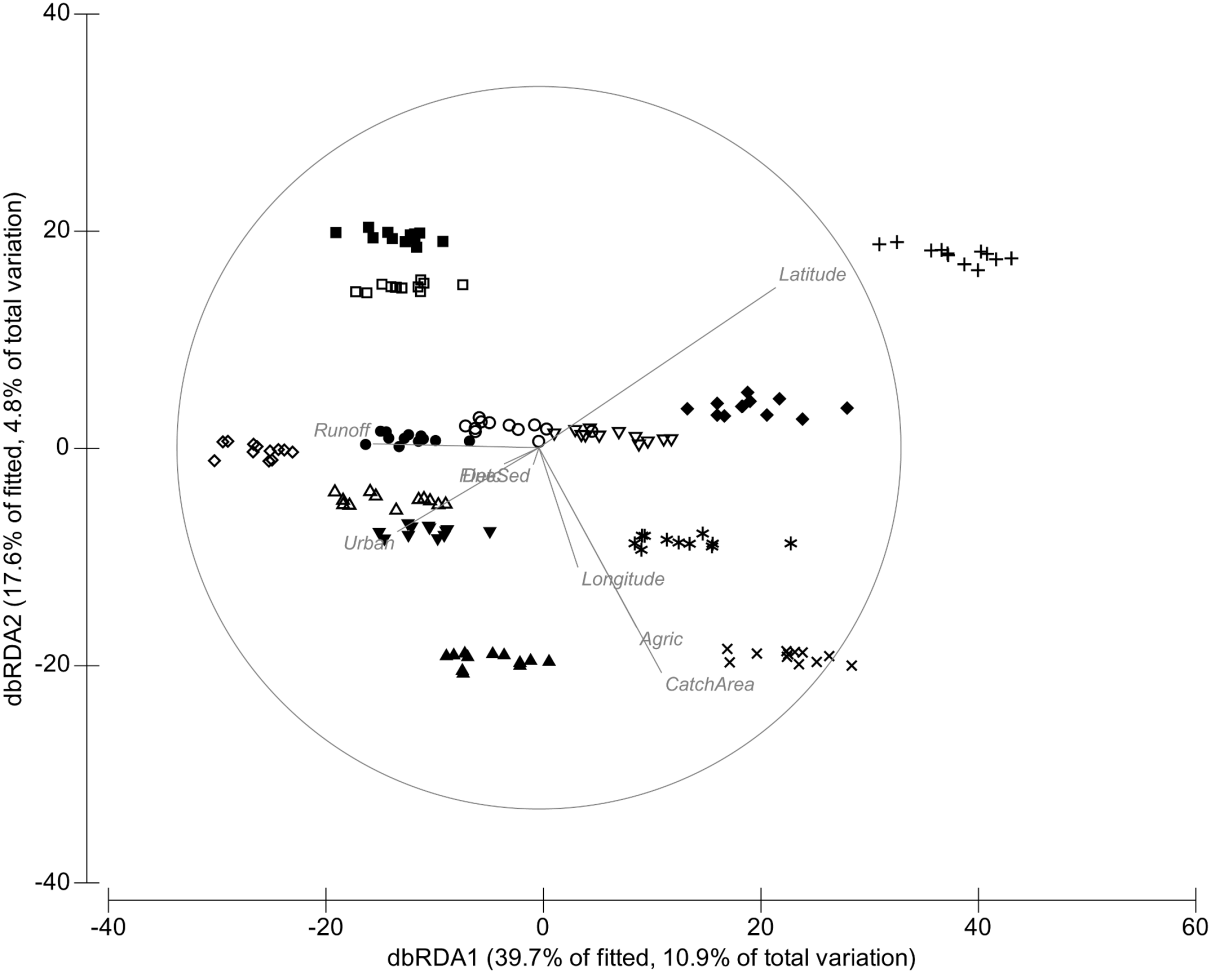
Distance-based redundancy analysis (dbRDA) of macroinvertebrate samples in spring. Distance-based redundancy analysis (dbRDA) of macroinvertebrate samples in spring, overlaid with normalised predictor variables (based on distLM analysis in Table 4). Abbreviations for predictor variables are listed in S1 Table.

Since multivariate analysis demonstrated that community structure differed between seasons (Table 1), we evaluated them separately for the remaining analyses. In autumn, 8 out of the 12 predictor variables explained significant amounts (total of 23.7%) of the variability in community composition (Table 4, Fig 3). Conductivity was most strongly related (explaining 6.8% of the total variation in the assemblage structure) to the community structure, followed by latitude (3.4%) and agriculture (3.1%). Macroinvertebrate communities among sites in autumn were more closely clustered together (Fig 3) relative to spring (Fig 4).

**Table 4:** Results from a distance-based linear model (DistLM) for the 13 sites in autumn and spring. Variables are listed in order of contribution to explaining variation in the community composition. % variation represents explained variation attributable to each variable added to the model. Abbreviations for predictor variables are listed in S1 Table.

Variable	*F*	*P*-value	% variation
**Autumn (*R*** ^***2***^ **= 23.7; AIC = 1275.9)**			
Cond	11.71	**< 0.001**	6.82
Latitude	5.93	**< 0.001**	3.35
Agric	5.55	**< 0.001**	3.05
Urban	5.03	**< 0.001**	2.69
CatchArea	4.97	**< 0.001**	2.60
Longitude	4.66	**< 0.001**	2.38
Detc	3.36	**< 0.001**	1.69
FineSed	2.33	**< 0.001**	1.16
**Spring (*R*** ^***2***^ **= 27.3; AIC = 1208.1)**			
Cond	14.66	**< 0.001**	8.74
Latitude	7.97	**< 0.001**	4.54
Longitude	5.94	**< 0.001**	3.28
Agric	5.01	**< 0.001**	2.70
CatchArea	4.99	**< 0.001**	2.61
Urban	3.83	**< 0.001**	1.97
Runoff	3.04	**< 0.001**	1.54
FineSed	2.83	**< 0.001**	1.41
Detc	1.82	**< 0.001**	1.41

In spring, 9 out of the 12 predictor variables explained significant amounts (total of 27.3%) of the variability in community composition (Table 4, Fig 4). Again, conductivity was most strongly related (8.7% of the total variation in the assemblage structure) to community structure, followed by latitude (4.5%) and longitude (3.3%). Sites were distinct in their community composition in spring (Fig 4) relative to autumn (Fig 3).

A number of variables (conductivity, latitude, agriculture and urban land-uses, catchment area, longitude, detritus cover and fine sediments) were consistently predictive of macroinvertebrate community structure in both seasons. Predictor variables explained more of the variation in community structure in spring than in autumn (Table 4).

Results from the univariate analyses showed that during autumn, richness was positively related with increased cover by agriculture and urban land-uses as well as detritus cover, but reduced with conductivity, longitude, and fine sediments (Table 5). Evenness was positively related with increased cover by only urban land-use (Table 5). Diversity was also positively related with increased cover by urban land-use and declined with conductivity. Urban land-use was a predictor across all 3 univariate measures used in the analysis. The stepwise regression models explained more of the variation in richness than evenness or diversity.

**Table 5:** Results of general linear models for the relationships of the biodiversity indices to geographic, environmental and land-use predictor variables in autumn and spring. S.E. represents the standard error of the coefficients. Bold numbers indicate significant P-values. *indicates trending *P*-values. Abbreviations for predictor variables are listed in S1 Table.

**Autumn**				**Spring**			
Variables	Coefficient ± S.E.	*t*- value	*P*-values	Variables	Coefficient ± S.E.	*t*- value	*P*-values
**Richness (*R*** ^**2**^ **= 0.46)**				**Richness (*R*** ^**2**^ **= 0.36)**			
Intercept	7.36 ± 1.72	4.29	**< 0.001**	Intercept	9.42 ± 1.16	8.13	**< 0.001**
Long	-0.68 ± 0.36	-1.92	*0.057	Long	-0.87 ± 0.38	-2.30	**0.023**
Agric	1.59 ± 0.56	2.84	**0.005**	Agric	1.44 ± 0.67	2.14	**0.034**
Urban	5.50 ± 1.35	4.09	**< 0.001**	Urban	6.29 ± 1.45	4.34	**< 0.001**
Cond	-1.02 ± 0.15	-6.78	**< 0.001**	Cond	-0.85 ± 0.16	-5.24	**< 0.001**
PH	0.38 ± 0.23	1.65	0.100				
FineSed	-0.63 ± 0.26	-2.42	**0.017**	**Evenness (*R*** ^**2**^ **= 0.17)**			
Detc	1.40 ± 0.43	3.27	**0.001**	Intercept	1.08 ± 0.16	6.68	**< 0.001**
				Lat	0.04 ± 0.02	1.84	*0.067
**Evenness (*R*** ^**2**^ **= 0.08)**				Runoff	-59.76 ± 35.38	-1.69	*0.093
Intercept	0.57 ± 0.02	29.55	**< 0.001**	Urban	0.26 ± 0.10	2.50	**0.014**
Urban	0.27 ± 0.07	3.80	**< 0.001**	Cond	-0.07 ± 0.02	-3.85	**< 0.001**
				FineSed	0.04 ± 0.03	1.70	*0.091
**Diversity (*R*** ^**2**^ **= 0.21)**							
Intercept	2.75 ± 0.31	8.80	**< 0.001**	**Diversity (*R*** ^**2**^ **= 0.26)**			
Urban	1.28 ± 0.29	4.35	**< 0.001**	Intercept	3.86 ± 0.51	7.56	**< 0.001**
Cond	-0.11 ± 0.04	-3.10	**0.002**	Lat	0.15 ± 0.08	2.06	**0.041**
				Urban	1.13 ± 0.36	3.14	**0.002**
				Cond	-0.29 ± 0.06	-4.59	**< 0.001**
				FineSed	0.14 ± 0.09	1.52	0.132

During spring, richness was positively related with increased cover by agriculture and urban land-uses but declined with longitude and conductivity. Evenness was positively related with increased urban land-use but negatively related with conductivity. Evenness exhibited trends of decline with runoff but exhibited increasing trends with fine sediment and latitude (Table 5). Diversity was positively related with increased cover by urban land-use and latitude but declined with conductivity. Urban land-use and conductivity were predictors across all three univariate biodiversity indices used in the analysis. The stepwise regression models explained more of the variation in richness than evenness or diversity (Table 5).

A small number of variables were consistently predictive of univariate indices in both seasons. Conductivity, agriculture and urban land-uses were significantly associated with richness in autumn and spring, but only urban land-use was predictive of evenness in both seasons. Conductivity and urban land-use were predictive of diversity in autumn and spring.

### Aim 3: Candidate taxa that correlate with gradients of particular land-use, environmental and geographic determinants

Out of a total of 840 macroinvertebrates taxa used in the analysis, 14 taxa were significantly correlated with the strongest predictors of community composition in autumn (Mantel’s test: *P* < 0.001, ρ = 0.43) (Table 6). Out of these 14 taxa, 13 were all negatively correlated with conductivity and 11 taxa were all negatively correlated with latitude. The abundance of Dixidae and *Physa acuta* was negatively correlated with catchment area (stream size) and latitude respectively. Only one taxon (*Aphroteniella* sp.) was correlated with gradient of conductivity, cover by agriculture and urban land-uses, catchment area, longitude and detritus cover. The abundance of *Aphroteniella* sp. was negatively correlated with agriculture and stream size but positively related to cover by urban land-use (Table 6).

**Table 6:** Macroinvertebrates indicated by BVSTEP as associated with gradients of specific predictor variables in autumn and spring. Numbers written in the cells are Spearman’s correlation values between the taxon and gradient of that predictor variable. Predictor variables for both seasons are arranged in the order in which the most influential variables in each season appears as indicated by DistLM (Table 4) appear. Abbreviations for predictor variables are listed in S1 Table.

**Autumn**											
Taxa	Family	Class/ Order	Cond	Lat	Agric	Urban	CatchArea	Long	Detc	FineSed	
**ρ**			0.50	0.47	0.35	0.30	0.35	0.35	0.10	0.14	
***P*-value**			**< 0.001**	**< 0.001**	**< 0.001**	**< 0.001**	**< 0.001**	**< 0.001**	0.151	**0.018**	
*Physa acuta*	Physidae	Gastropoda	-0.3	-0.5							
*Nais sp*.	Naididae	Oligochaeta	-0.4	-0.3							
Chaetogaster	Naididae	Oligochaeta	-0.2	-0.3							
Tipulidae	Tipulidae	Diptera	-0.2	-0.3						0.2	
Dixidae	Dixidae	Diptera	-0.3	-0.1	-0.4		-0.5	1x10^-9^			
*Aphroteniella sp*.	Aphroteniinae	Diptera	-0.4	1x10^-12^	-0.4	0.4	-0.4	0.1	-0.1		
*Riethia sp*.	Pseudochironomini	Oligochaeta	-0.3	-0.2							
*Tasmanocoenis tillyardi*	Caenidae	Ephemeroptera	-0.4	-0.2							
*Sigara sp*.	Corixidae	Hemiptera	-0.2	-0.3							
*Newmanoperla thoreyi*	Gripopterygidae	Plecoptera	-0.2								
*Hellyethira simplex*	Hydroptilidae	Trichoptera	-0.3	-0.3							
*Oxyethira columba*	Hydroptilidae	Trichoptera	-0.2	-0.2							
*Lingora aurata*	Conoesucidae	Trichoptera									
*Leptorussa sp*.	Leptoceridae	Trichoptera	-0.2	-0.3						0.2	
**Spring**											
Taxa	Family	Order	Cond	Lat	Long	Agric	CatchArea	Urban	Runoff	FineSed	Detc
**ρ**			0.44	0.51	0.36	0.31	0.27	0.35	0.36	0.19	0.16
***P*-value**			**< 0.001**	**< 0.001**	**< 0.001**	**< 0.001**	**< 0.001**	**< 0.001**	**< 0.001**	**0.004**	**0.009**
*Ferrissia petterdi*	Ancylidae	Gastropoda		-0.3					0.2		
*Physa acuta*	Physidae	Gastropoda	-0.2	-0.3					0.3		
*Nais sp*.	Naididae	Oligochaeta		-0.1					0.4		
*Paranais litoralis*	Naididae	Oligochaeta									
*Gammarus sp*.	Eusiridae	Amphipoda									
Eusiridae	Eusiridae	Amphipoda		0.3							
Perthiidae	Perthiidae	Amphipoda						0.3			
*Necterosoma penicillatus*	Dytiscidae	Coleoptera		0.4				-0.3			
Dixidae	Dixidae	Diptera			1x10^-16^						
Empididae	Empididae	Diptera	-0.4								
*Larsia sp*.	Chironomidae	Diptera						-0.3			
*Corynoneura sp*.	Orthocladiinae	Diptera							0.4		
*Riethia sp*.	Pseudochironomini	Oligochaeta	-0.4	-0.3							
*Stempellina sp*.	Chironominae	Diptera		-0.3							
Leptophlebiidae	Leptophlebiidae	Ephemeroptera	-0.4	-0.3					0.5		
*Tasmanocoenis tillyardi*	Caenidae	Ephemeroptera	-0.4	-0.2					0.4		
*Micronecta sp*.	Corixidae	Hemiptera									-0.3
*Anisops sp*.	Notonectidae	Hemiptera						-0.3			
*Austrolestes annulosus*	Lestidae	Odonata									0.2
*Diplacodes haematodes*	Libellulidae	Odonata		0.3							
*Orthetrum caledonicum*	Libellulidae	Odonata									
Libellulidae	Libellulidae	Odonata		0.3							
*Newmanoperla thoreyi*	Gripopterygidae	Plecoptera	-0.4			-0.5			0.3		
Gripopterygidae	Gripopterygidae	Plecoptera	-0.3								0.1
*Austrocerca tasmanica*	Notonemouridae	Plecoptera	-0.4	-0.2	-0.1				0.5		
Hydrobiosidae	Hydrobiosidae	Trichoptera									
*Oxyethira columba*	Hydroptilidae	Trichoptera	-0.3	-0.4	-0.4	-0.4	0.1	-0.4		0.3	
*Lingora aurata*	Conoesucidae	Trichoptera									
*Atriplectides dubius*	Atriplectidae	Trichoptera									0.1
*Lectrides varians*	Leptoceridae	Trichoptera	-0.5						0.4		
*Leptorussa sp*.	Leptoceridae	Trichoptera						-0.2			
*Notalina bifaria*	Leptoceridae	Trichoptera									1x10^-11^

ρ signifies Spearman’s correlations and *P*-value shows the significance of the relationship between the macroinvertebrates and the predictor variables. Blank cells between taxon and predictor variable indicate that taxon was not correlated with that predictor variable.

A number of taxa were also weakly (|-0.4 ≤ ρ ≤ -0.1|; |0.1 ≤ ρ ≤ 0.4|) and moderately (ρ = 0.5, ρ = -0.5) correlated with the predictor variables (Table 6). Taxa that correlated with more than one predictor variables were common.

In spring, 32 taxa were correlated (Mantel’s test: *P* < 0.001, ρ = 0.59) with the most related predictors of macroinvertebrate community composition (Table 6). Ten of these taxa were significantly correlated with conductivity. The abundance of all these taxa declined with increasing conductivity. A total of 9 taxa were correlated with runoff. The abundance of all these taxa increased with increasing runoff. Two and 6 taxa were significantly correlated with agriculture and urban land-uses respectively. The abundance of *Newmanoperla thoreyi* was negatively correlated with cover by agriculture land-use (Table 6). A number of taxa were weakly (|-0.4 ≤ ρ ≤ -0.1|; |0.1 ≤ ρ ≤ 0.4|) and moderately (*ρ* = 0.5, ρ = -0.5) correlated with the predictor variables. 'Taxa correlated with greater than one predictor variables were common. Nine taxa were common to both seasons.

## Discussion

Using a 13 year dataset, we found the following: (1) temporal trajectories of macroinvertebrate communities in temporary streams varied within sites in both seasons and across the years. Temporal trajectories of macroinvertebrate communities differed between sites but there was no consistent trend in the trajectories within sites across years; (2) a combination of land-use, geographic and environmental variables accounted for 24% of the variation in the community structure in autumn and 27% in spring; (3) in autumn 14 taxa were significantly related to the most related predictors of community structure across sites. In contrast, during spring, 32 taxa were significantly related to the most related predictors of community structure. Our results indicate that temporal variability of macroinvertebrates in these temporary streams is predicted significantly (but modestly) by a combination of factors but most strongly and consistently related to conductivity, longitude, latitude and the proportion of catchment under agricultural and urban land-uses.

### Temporal changes in macroinvertebrates composition

Macroinvertebrate community composition varied among individual sites across the years. Given the variability in the biophysical variables among years, this result was unsurprising. These differences may also have been observed because of differences in the ability for macroinvertebrates to have survived at each site [40]. Similarly, a study by Leigh and Sheldon (5] also found high temporal variability of macroinvertebrates in Australian dryland rivers. Our results may indicate that stochastic processes such as climate variability or differential dispersal abilities of macroinvertebrates may be important [41]. Community composition also varied seasonally within sites. Sites in autumn were more similar in their community composition than sites in spring. The strongest correlates of community structure in autumn included all the predictor variables used in the analysis except runoff, fine sediments, algal cover, dissolved oxygen and pH, whereas in spring fine sediment and runoff were also related to community structure. The differences in community composition between seasons may be due to the fact that different taxa show differential success between seasons according to their particular resilience or resistance traits [41].

We predicted that during summer (characterized by high temperature, little or no flows), macroinvertebrates diversity would decline relative to wetter, cooler spring conditions. However, we found that within the same site, diversity and evenness were significantly higher in autumn than in spring whereas richness did not vary between the two seasons. This distinction in diversity and richness among the two seasons could also be due to the combination of the multiple physical factors and the inherent flow variability that characterize temporary streams.

### Relationship of macroinvertebrate community composition, environmental, geographic and land-use predictor variables

Our study has shown that conductivity was the most consistent predictor of assemblage composition in both autumn and spring. Conductivity alone was associated with more variation in macroinvertebrate assemblage structure than any other land-use, geographic or environmental variable. These responses are reflected in the declines in richness, evenness and diversity with conductivity in both seasons. The relationship is broadly consistent with earlier studies describing salinity as a major driver of community composition [42–44]. We therefore propose that salinity exerts a strong direct pressure on macroinvertebrate assemblages in temporary streams by selecting for saline-tolerant taxa, while selecting against the more halo-sensitive taxa, thus leading to general declines in richness, evenness and diversity.

Our results showed that, in autumn, macroinvertebrate communities among sites in these temporary streams were more closely clustered together relative to spring, suggesting that community composition was more similar in autumn than in spring. This difference in assemblage composition between the two seasons may be likely due to differences in flow variability that characterize these temporary streams [45]. Our study showed that agriculture and urban land-uses were also significant predictors of community structure in autumn and spring, with agriculture being the most related land-use predictor. Richness, evenness and diversity were strongly correlated with the land-use variables in both seasons. These responses were reflected in the declines in richness, evenness and diversity with agricultural land-use. These relationships were broadly consistent with earlier studies describing changes in macroinvertebrate communities in agricultural catchments [36, 46]. These patterns may be driven by multiple mechanisms common to all agricultural land-use [14], such as changes in water quality (including enrichment of nutrients and increases in salinity and temperature), lack of riparian zones and dominance of fine sediments [36, 46, 47].

Our results showed that geographic location variables (latitude and longitude) were predictive of community structure in both autumn and spring. To some extent, decreasing latitude in this region is correlated with increasing dryness and increasing salinity (see S2 Table), while Longitude may reflect the rain-shadow effect of the Mount Lofty Ranges in part of the study region. Richness, evenness and diversity were significantly correlated with the location variables in both seasons. These relationships were broadly consistent with earlier studies describing spatial variability of macroinvertebrate communities among different sites (locations) [48, 49]. Our results indicate that understanding biogeography of community structure is important for conservation because different sites at large spatial scales harbor different components of the regional assemblage and this variation is not captured by considering conductivity alone.

### Candidate taxa that correlated with gradients of particular land-uses, environmental and geographic variables

When streams are disturbed, taxa that are sensitive to those stressors will be eliminated, leaving communities to be dominated by only taxa that are resistant (able to survive the impacts) or resilient (have efficient recovery mechanisms). Our results showed that in both seasons, a number of taxa were weakly to moderately correlated with the strongest correlates of community structure. The reason for these weak to moderate correlations might be due to the sparseness of most taxa recorded and the finer taxonomic resolution (genus and species) we used in our analysis. Furthermore, it was common that a taxon, which was correlated with a single predictor variable, also responded significantly to other predictor variables. This may be due to the autocorrelations that existed among the predictor variables used in our analysis. Underwood and Peterson [50] described indicator taxa as those taxa that are highly correlated with a predictor variable of interest and not correlated with any other predictor variable. Under this definition, no indicator taxa were evident in autumn and 10 indicator taxa (Perthiidae, Empididae, *Corynoneura sp*., *Micronecta sp*., *Anisops sp*., *Austrolestes annulosus*, *Diplacodes haematodes*, Libellulidae, *Atriplectides dubius*, *Leptorussa sp*.) were recorded in spring. Since these “indicator taxa” provided a poor representation of the overall variability (and were also weakly correlated with the predictor variables of interest), alternative approaches to identifying indicators (e.g. trait-based approaches) [51] may provide additional information useful for condition assessment in temporary streams.

## Conclusions

When temporary streams become impacted with harsh natural or anthropogenic conditions, macroinvertebrate communities tend to become more similar because the tolerant macroinvertebrate generalists dominate [52]. The differential colonization and survival of macroinvertebrates in this study highlights the importance of local factors in structuring macroinvertebrate communities, particularly conductivity, location, and the extent of agricultural and urban land-uses. The highly variable nature of temporary streams, coupled with the site-specific changes in macroinvertebrate assemblages, pose a challenge when developing monitoring programmes and managing such waters [52]. The effects of anthropogenic degradation may mimic natural declines in species abundance and diversity, which are related to seasonal recession of temporary streams, creating difficulties in separating changes due to human impact from those due to natural processes. Although we provide an improved understanding of the temporal variability in assemblage composition of intermittent streams, the extreme variability we found using taxonomically-based metrics presents an even more challenging scenario for monitoring. Alternative approaches to biomonitoring using traits may provide additional information useful for measures of the conditions in temporary streams [9, 53].

## Supporting Information

S1 Table List of environmental, geographic and land use predictor variables. (DOCX)

S2 Table Spearman’s correlations coefficients (ρ) between environmental, geographic and land-use predictor variables for the 13 sites. Abbreviations for predictor variables are listed in S1 Table. Bold numbers indicate ρ > 0.90 between variables for which reason one variable for chosen as a surrogate for the other variable.
(DOCX)

S3 Table List of 13 sites surveyed in this study. (DOCX)
