# Supplementary material for: Temporal Patterns and Environmental Correlates of Macroinvertebrate Communities in Temporary Streams
Source: PLoS One. 2015 Nov 10;10(11):e0142370. doi: 10.1371/journal.pone.0142370 (PMC4640519; doi:10.1371/journal.pone.0142370)
Supplement: S1 Table — (DOCX) [file pone.0142370.s001.docx]

**Table S1: Summary statistics for the environmental, geographic and land use predictor variables among the study system.** Min: minimum value; Max: maximum value; sd: standard deviation.

| **Predictor variable** | **Constituent (%)** | **Symbol** | **Min** | **Max** | **Mean** | **sd** |
| --- | --- | --- | --- | --- | --- | --- |
| ***Environmental variable*** |  |  |  |  |  |  |
| Fine sediment score (%) | Sand + Silt + Clay | FineSed | 0 | 100 | 59.54 | 36.55 |
| Algal cover score (%) |  | Algc | 0 | 60 | 3.58 | 8.58 |
| Detritus cover score (%) |  | Detc | 0 | 100 | 30.55 | 24.12 |
| pH |  | pH | 5.63 | 8.73 | 7.70 | 0.58 |
| Dissolved oxygen (mg/L) |  | DO | 2.80 | 19.82 | 9.33 | 2.72 |
| Conductivity (µS/cm) |  | Cond | 139 | 23700 | 3709 | 4343 |
| Local discharge (Runoff + drainage) (mm /day) |  | Runoff | 4.7 x 10^-6^ | 2.3 x 10^-3^ | 0.0003 | 0.0004 |
| ***Geographic variables*** |  |  |  |  |  |  |
| Latitude (decimal degrees) |  | Lat | -35.95117 | -32.096108 |  |  |
| Longitude (decimal degrees) |  | Long | 136.708832 | 139.364501 |  |  |
| Catchment area above sample site (km^2^) |  | CatchArea | 9.18 | 826.37 | 221.72 | 220.98 |
| Distance from source (km) |  | DFS | 3.5 | 83.1 | 31.96 | 20.66 |
| ***Land use variables based on ALUM categories (% area of upstream catchment)*** |  |  |  |  |  |  |
| Conservation and minimal use (%) | Nature conservation + Other minimal uses | ConsvMin | 0 | 98.23 | 21.78 | 30.09 |
| Agriculture (%) | Cropping + Grazing modified pastures + Grazing natural vegetation + Intensive animal production + Intensive horticulture + Irrigated cropping + Irrigated modified pastures + Irrigated perennial horticulture + Irrigated plantation forestry + Irrigated seasonal horticulture + Perennial horticulture + Plantation forestry | Agric | 0.31 | 98.77 | 69.66 | 33.98 |
| Urban (%) | Residential + Transport and Communication | Urban | 0.64 | 32.60 | 6.90 | 8.50 |
