# Supplementary material for: Temporal Patterns and Environmental Correlates of Macroinvertebrate Communities in Temporary Streams
Source: PLoS One. 2015 Nov 10;10(11):e0142370. doi: 10.1371/journal.pone.0142370 (PMC4640519; doi:10.1371/journal.pone.0142370)
Supplement: S2 Table — Abbreviations for predictor variables are listed in S1 Table. Bold numbers indicate ρ > 0.90 between variables for which reason one variable for chosen as a surrogate for the other variable. (DOCX) [file pone.0142370.s002.docx]

**Table S2:** Spearman’s correlations coefficients (ρ) between environmental, geographic and land-use predictor variables for the 13 sites.

|  | **Runoff** | **Agric** | **ConsvMin** | **Urban** | **CatchArea** | **DFS** | **Lat** | **Long** | **Cond** | **DO** | **pH** | **FineSed** | **Algc** |
| --- | --- | --- | --- | --- | --- | --- | --- | --- | --- | --- | --- | --- | --- |
| **Runoff** |  |  |  |  |  |  |  |  |  |  |  |  |  |
| **Agric** | -0.24 |  |  |  |  |  |  |  |  |  |  |  |  |
| **ConsvMin** | 0.18 | **-0.97** |  |  |  |  |  |  |  |  |  |  |  |
| **Urban** | 0.33 | -0.44 | 0.23 |  |  |  |  |  |  |  |  |  |  |
| **CatchArea** | -0.21 | 0.36 | -0.32 | -0.25 |  |  |  |  |  |  |  |  |  |
| **DFS** | -0.25 | 0.45 | -0.39 | -0.39 | **0.96** |  |  |  |  |  |  |  |  |
| **Lat** | -0.07 | 0.57 | -0.59 | -0.29 | 0.08 | 0.11 |  |  |  |  |  |  |  |
| **Long** | -0.04 | 0.57 | -0.67 | -0.28 | 0.16 | 0.25 | 0.18 |  |  |  |  |  |  |
| **Cond** | -0.20 | 0.69 | -0.68 | -0.35 | 0.48 | 0.55 | 0.68 | 0.34 |  |  |  |  |  |
| **DO** | -0.06 | 0.13 | -0.05 | -0.13 | 0.01 | 0.02 | 0.30 | 0.02 | 0.20 |  |  |  |  |
| **pH** | -0.001 | 0.32 | -0.38 | -0.04 | 0.21 | 0.22 | -0.40 | 0.32 | 0.49 | 0.25 |  |  |  |
| **FineSed** | 0.05 | -0.22 | 0.16 | 0.13 | -0.09 | 0.00 | -0.18 | -0.31 | -0.10 | -0.22 | -0.20 |  |  |
| **Algc** | -0.05 | 0.23 | -0.15 | -0.10 | 0.11 | 0.09 | 0.16 | 0.17 | 0.15 | 0.10 | 0.18 | -0.26 |  |
| **Detc** | 0.09 | -0.24 | 0.11 | 0.19 | -0.09 | -0.03 | -0.21 | -0.08 | -0.18 | -0.21 | -0.21 | 0.41 | -0.21 |

Abbreviations for predictor variables are listed in Table S1. Bold numbers indicate ρ > 0.90 between variables for which reason one variable for chosen as a surrogate for the other variable.
