# Supplementary material for: Temporal Patterns and Environmental Correlates of Macroinvertebrate Communities in Temporary Streams
Source: PLoS One. 2015 Nov 10;10(11):e0142370. doi: 10.1371/journal.pone.0142370 (PMC4640519; doi:10.1371/journal.pone.0142370)
Supplement: S3 Table — (DOCX) [file pone.0142370.s003.docx]

Table S3 List of 13 sites surveyed in this study

| Site | Symbol | Latitude | Longitude |
| --- | --- | --- | --- |
| Bremer river | ▲ | -35.169208 | 139.021567 |
| Finniss river | ▲ | -35.322908 | 138.66722 |
| First creek | ■ | -34.971108 | 138.678886 |
| Hill river | ♦ | -33.616457 | 138.62965 |
| Hindmarsh river | ● | -35.468682 | 138.585333 |
| Kanyaka creek | + | -32.096108 | 138.291572 |
| Light river | × | -34.358319 | 138.972061 |
| Marne river | * | -34.653859 | 139.364501 |
| Myponga river | ∆ | -35.382156 | 138.475646 |
| North Para river | ∇ | -34.46357 | 139.040276 |
| Rocky river | □ | -35.95117 | 136.708832 |
| Scott creek | ◊ | -35.099448 | 138.67251 |
| Torrens river | ○ | -34.795666 | 139.003485 |
